# Traffic-Related Air Pollution and the Onset of Myocardial Infarction: Disclosing Benzene as a Trigger? A Small-Area Case-Crossover Study

**DOI:** 10.1371/journal.pone.0100307

**Published:** 2014-06-16

**Authors:** Denis Bard, Wahida Kihal, Charles Schillinger, Christophe Fermanian, Claire Ségala, Sophie Glorion, Dominique Arveiler, Christiane Weber

**Affiliations:** 1 Department of Epidemiology and Biostatistics, École des Hautes Études en Santé Publique, Rennes and Sorbonne Paris Cité, Paris, France; 2 Association pour la Surveillance de la Qualité de l'Air en Alsace-ASPA, Schiltigheim, France; 3 SEPIA-Santé, Baud, France; 4 Department of Epidemiology and Public Health (EA3430), University of Strasbourg, Strasbourg, France; 5 Laboratoire Image, Ville, Environnement (LIVE UMR7362 CNRS), Faculté de géographie et d'aménagement, University of Strasbourg, Strasbourg, France; University of Tennessee Health Science Center, United States of America

## Abstract

**Background and Objectives:**

Exposure to traffic is an established risk factor for the triggering of myocardial infarction (MI). Particulate matter, mainly emitted by diesel vehicles, appears to be the most important stressor. However, the possible influence of benzene from gasoline-fueled cars has not been explored so far.

**Methods and Results:**

We conducted a case-crossover study from 2,134 MI cases recorded by the local Coronary Heart Disease Registry (2000–2007) in the Strasbourg Metropolitan Area (France). Available individual data were age, gender, previous history of ischemic heart disease and address of residence at the time of the event. Nitrogen dioxide, particles of median aerodynamic diameter <10 µm (PM_10_), ozone, carbon monoxide and benzene air concentrations were modeled on an hourly basis at the census block level over the study period using the deterministic ADMS-Urban air dispersion model. Model input data were emissions inventories, background pollution measurements, and meteorological data. We have found a positive, statistically significant association between concentrations of benzene and the onset of MI: per cent increase in risk for a 1 µg/m^3^ increase in benzene concentration in the previous 0, 0–1 and 1 day was 10.4 (95% confidence interval 3–18.2), 10.7 (2.7–19.2) and 7.2 (0.3–14.5), respectively. The associations between the other pollutants and outcome were much lower and in accordance with the literature.

**Conclusion:**

We have observed that benzene in ambient air is strongly associated with the triggering of MI. This novel finding needs confirmation. If so, this would mean that not only diesel vehicles, the main particulate matter emitters, but also gasoline-fueled cars –main benzene emitters–, should be taken into account for public health action.

## Introduction

The effects of traffic-related air pollution on cardiorespiratory mortality have been consistently established since the late 1980s [1]. Further studies specifically investigated the association between exposure to traffic and the onset of myocardial infarction (MI), one of the most frequent causes of death. Since the publication of the seminal paper by Peters *et al*. (2001) [2], which shows an association between exposure to traffic and the onset of myocardial infarction (MI), many studies have been published on the issue. The latest review and meta-analysis to date considered the association between short-term exposure to traffic-related air pollutants and subsequent MI risk [3]. The authors retained 34 studies, considering the effects of various air pollutants, either alone or in association, *e.g*. particles with aerodynamic diameter <10 µm (PM_10_), particles with diameter <2.5 µm (PM_2.5_), black carbon/black smoke, ozone (O_3_), carbon monoxide (CO), nitrogen oxides and sulphur dioxide. Using a random-effect model to estimate the meta-relative risk and 95% confidence interval, a significant, positive association appeared between all analyzed pollutants, with the exception of ozone, and MI risk, although published studies are quite inconsistent regarding both the direction of the association and statistical significance for all pollutants. Thus, the present state of knowledge strongly supports the role of exposure to traffic-related air pollution in triggering the onset of MI. In addition, PM_10_ is the air pollutant most consistently associated with myocardial infarction onset [4], [5].

None of the above reviews mentions benzene, although gasoline- fueled engines emit this pollutant [6], [7]. The literature addressing the acute cardiovascular effects of benzene in occupational settings is scarce, *e.g*. [8], [9] but lends some support to an association between exposure to benzene and arrhythmias. However, in the Kotseva and Popov (1998) paper, benzene concentrations seem to have been very high (up to 65 mg/m^3^). The authors provide few details on the study population. In addition, probable co-exposures to various stressors are poorly discussed. To our knowledge, a single group investigated the cardiovascular effects of traffic-related air pollution in a mortality study in Taiwan, addressing exposure to benzene [10]. The authors found a significant association between benzene concentration and cardiovascular mortality (ICD-9-CM 410–411, 414, 430–437, same-day association: lag 0). However, this study considered only fatal cases of cardiovascular diseases. Furthermore, exposure was loosely defined on an ecological basis (air concentrations were measured at a single monitoring station for a study population defined as those living within a 10 km radius). Thus, the potential for error in exposure assessment was high.

The aim of the present study is to investigate the possible association between traffic-related benzene emissions as well as ‘classical’ traffic-related pollutants (NO_2_, PM_10_, O_3_, CO, SO_2_) and the onset of myocardial infarction. Study design was time-stratified case-crossover, using a very small area as the statistical unit.

## Methods

### Setting

The Strasbourg Metropolitan Area (SMA), an urban area of 28 municipalities (316 km^2^), is located in the Bas-Rhin district in northeastern France, with a population of about 450,000 inhabitants. It is subdivided into 190 census blocks of average population 2,000 (range 2–4,885) and a median area of 0.45 km^2^ (range 0.05–19.60). These blocks are the smallest administrative geographic unit in France for which socioeconomic and demographic information from the national census is available. They are devised as to be homogeneous for population size, socioeconomic characteristics and land use. Sixteen blocks of population size <250 were excluded of the dataset (0.8% of the SMA population), for the sake of compliance with French confidentiality regulations.

### Cases

Cases were all MI events (ICD-9: 410) either fatal or non-fatal, occurring in the age group 35–74 years between January 1, 2000 and December 31, 2007, ascertained by the local Bas-Rhin Coronary Heart Disease, a collaborating center of the WHO MONICA Project [11]. MIs were documented events, which have definitively been diagnosed as such whether clinically or at necropsy. Individual data available were age, gender, previous history of ischemic heart disease, and address of residence at the time of the event. Cases were geocoded to their census block of residence using ArcGIS version 9.1 (ESRI, Redlands, CA).

### Assessment of exposure to air pollution

Nitrogen dioxide (NO_2_), particles of median aerodynamic diameter <10 µm (PM_10_), ozone (O_3_), carbon monoxide (CO), sulphur dioxide (SO_2_) and benzene air concentrations were modeled on an hourly basis at the census block level over the whole study period using the deterministic ADMS-Urban air dispersion model (Atmospheric Dispersion Modeling System) [12]. Model input data comprised of emissions inventories, background pollution measurements and meteorological data. More details can be found elsewhere [13]. Model performance assessment took place on two occasions. First, we compared predictions to measures at monitoring stations on a yearly basis for NO_2_, O_3_, and SO_2_. However, there was no routine station measurement for CO. Mean differences between measured and modeled values were −2% (range −10% to 9%) for NO_2_, −4% (−10% to 10%) for O_3_, −1% (−2% to 8%) for PM_10_. Second, we used passive samplers to measure benzene, PM_10_, and NO_2_ concentrations at the census block level (61 measurements points for NO_2_ and benzene, four occasions throughout the year, seven points for PM_10_ on eight occasions). Measurement points were selected as to cover the SMA and compared to the predicted value for the census block, both on a yearly and hourly basis. For both circumstances and whatever the pollutant, the mean error was small: −1% (range −39% to 42%) for NO_2_, 0% (−26% to 11%) for PM_10_, −10% (−33% to 30%) for benzene. One exception was SO_2_, of which concentrations were low (maximum 11 µg/m^3^), and modeling performance was poor. Accordingly, we did not consider SO_2_ further in the analysis.

### Proven or likely confounders

Daily meteorological variables (temperature, atmospheric pressure, and relative humidity) were obtained from the French meteorological service (Météo France); weekly influenza-like case counts came from the Sentinelles network [14] of the French National Institute of Health and Medical Research.

### Statistical analysis

Associations between MI events and air pollution were assessed with a case-crossover model [15]. Control days were defined according to a monthly time-stratified design [16]. For a MI occurring on a given weekday (*e.g*., a Monday), control days were the same days of the week throughout the rest of the month (thus, three or four days; here, the other Mondays of the month). Associations between MI events and ambient air pollution concentrations modeled by census block were estimated, adjusting for holidays, meteorological variables (daily maximum temperature, maximum atmospheric pressure, and mean relative humidity), and influenza epidemics. We tested the influence of the various lags reported in the literature between air pollution indicators, treated as continuous variables, and MI events: average of the day of the event: (lag 0), average of the day of the event and the 1^st^ previous day (lag 0–1), and average of the previous day (lag 1).

The daily air pollution indicator considered for NO_2_, PM_10_, CO and benzene was the 24-hour average concentration, and for ozone, it was the maximum daily value of the 8-hour moving average. The analysis for ozone considered MI events occurring between April 1 and September 30 of each year, because of the very low concentrations of this pollutant in winter. Associations were assessed for cases of all ages and then for cases aged 35–54 and 55–74 years, respectively (categorizing was only for these two age groups, for ensuring confidentiality of the health data geocoded by census blocks).

We employed conditional logistic regression for analyses. Results are expressed as the percent increase in MI risk per 10 µg/m^3^ increase in pollutant concentrations for NO_2,_ PM_10_ and O_3_, per 100 µg/m^3^ increase for CO, and per 1 µg/m^3^ increase for benzene. All statistical analyses were performed with SAS 9.1 software (SAS Institute, Cary, NC).

### Ethics statement

The French data protection authority (CNIL) approved the study.

## Results

Over the 8 years of the study period, the Bas-Rhin Coronary Heart Disease Register ascertained 2,141 MI events. Seven (0.3%) events could not be geocoded and were excluded from the analyses. Thus, 2,134 cases were analyzed (Table 1), among whom 21.9% of females and 24.0% of males had a previous history of ischemic disease (ICD-9: 410–414). The small number of subjects with a previous history of ischemic disease precluded a specific analysis of this group, because of a very limited statistical power.

**Table 1:** Myocardial infarction events (ICD-9: 410) collected by the Bas-Rhin Coronary Heart Disease Register, Strasbourg Metropolitan Area, France, 2000–2007.

	Females (n = 492)	Males (n = 1,642)	Total (N = 2,134)
Age group	n (%)	n (%)	n (%)
35–54	136 (27.7)	637 (38.8)	773 (36.2)
55–74	356 (72.3)	1,005 (61.2)	1,361 (63.8)

Air pollution data appear in Table 2. The mean and range of air pollutants concentrations in the SMA for the study period were comparable to those observed in many metropolitan areas of Europe [17] or in the US [18]. All these pollutants were well correlated, as expected: Pearson's *r* for daily mean concentrations (Table 3) were in the range reported in the literature, the lowest being −0.16 (O_3_ and PM_10_) and the highest being 0.73 (NO_2_ and PM_10_), all statistically significant, in agreement with results reported in the literature [19], [20].

**Table 2:** Air pollutants daily concentrations (µg/m^3^) and meteorological parameters in the Strasbourg (France) Metropolitan Area, 2000–2007.

Pollutant	Mean	SD	Minimum	Q1	Median	Q3	Maximum
NO_2_	33.4	13.45	2.6	23.3	32.4	42.5	120.2
O_3_	63.3	36.85	1.1	35.45	59.41	85.0	228.3
PM_10_	21.1	9.94	1.65	14.1	19.3	26.1	107.5
CO	596.7	83.5	501.1	540.4	573.5	626.8	1800.5
Benzene	1.8	1.1	0.1	0.9	1.5	2.4	19.6
Minimum temperature (°C)	7.1	-	−15.3	2.0	7.5	12.5	21.8
Maximum atmospheric pressure (hPa)	1007.9	-	970.3	999.4	1005.9	1016.2	1043.1

**Table 3:** Ambient air pollution daily mean value correlation coefficients, Strasbourg (France) Metropolitan Area, 2000–2007.

	Pearson's *r*				
Pollutants	Benzene	NO_2_	O_3_	PM_10_	CO
Benzene	1.00				
NO_2_	0.64	1.00			
O_3_	−0.51	−0.34	1.00		
PM_10_	0.63	0.73	−0.16	1.00	
CO	0.60	0.72	−0.34	0.54	1.00

We have found a positive, statistically significant association between incremental concentrations of benzene and the onset of MI for our study population (base model), for all lags tested (0, 0–1 and 1 day), slightly more marked for the first two lags studied (Table 4). The associations between the other individual pollutants and outcome were essentially inconclusive (although a negative, statistically significant association appeared for ozone, lag 1).

**Table 4:** Exposure to air pollution and the onset of a myocardial infarction (MI) in the Strasbourg (France) Metropolitan Area, 2000–2007, base model^a^.

	Lag 0		Lag 0–1		Lag 1	
Pollutant	eOR (95%CI)	p value	eOR (95%CI)	p value	eOR (95%CI)	p value
Benzene	10.4 (3.0, 18.2)	0.005	10.7 (2.7, 19.·2)	0.008	7.2 (0.3, 14.5)	0.04
PM_10_	2.6 (−2.7, 8.2)		3.5 (−2.3, 9.7)		3.1 (−2.0, 8.5)	
NO_2_	4.7 (−0.2, 9.9)	0.06	5.4 (−0.1, 11.2)	0.05	3.6 (−1.0, 8.5)	
CO	3.2 (−6.1, 13.3)		4.4 (−6.6, 16.7)		3.0 (−6.2, 13.1)	
O_3_	−1.3 (−3.8, 1.3)		−2.7 (−5.5, 0.2)	0.07	**−3.1 (−5.7, −0.5)**	**0.02**

a Associations observed for different lag times; excess odds ratios (eOR) are expressed as per cent (95% confidence interval) increase for *i*) a 1 µg/m^3^ increase in benzene concentrations; *ii*) a 10 µg/m^3^ in NO_2_, O_3_ and PM_10_ concentrations and *iii*) a 100 µg/m3 increase in CO concentrations. Adjusted for the previous day maximum atmospheric pressure, same day minimum temperature and influenza epidemics.

When examining the risk (excess odds ratio –eOR–, that is, per cent increase in risk for a 1 µg/m^3^ increase in benzene concentration) associated to incremental exposure to benzene in specific population segments, we observed that men aged 35–54 years were particularly at risk (lag 0, eOR = 15.3; 95% confidence interval [1.0–31.7]), as was the case for older women (age group 55–74 years) for all lags, all statistically significant but more marked for lag 0–1 (eOR = 29.6 [10.3–52.2]) (Table 5).

**Table 5:** Exposure to air pollution and the onset of a myocardial infarction in the Strasbourg (France) Metropolitan Area, 2000–2007, by subgroups^a^.

		Lag 0	Lag 0–1	Lag 1
Gender (age group)	Pollutant	eOR (95% CI)	eOR (95% CI)	eOR (95% CI)
Males (35–54)	Benzene	15.3 (1.0, 31.7)*	11.1 (−3.5, 27.9)	3.8 (−8.2, 17.4)
	PM_10_	1.1 (−8.4, 11.6)	1.3 (−8.9, 12.6)	1.0 (−8.0, 10.9)
	NO_2_	9.3 (0, 19.4)*	7.0 (−2.9, 17.8)	2.1 (−6.0, 10.9)
	CO	10.3 (−7.6, 31.7)	4.4 (−15.0, 28.1)	−3.0 (−18.1, 14.9)
	O_3_	−0.4 (−4.9, 4·3)	−2.1 (−7.0, 3.2)	−3.0 (−7.5, 1.7)
Males (55–74)	Benzene	6.6 (−3.7, 18.0)	5.3 (−5.9, 17.8)	2.2 (−7.7, 13.1)
	PM_10_	3.8 (−4.1, 12.2)	3.7 (−4.9, 13.0)	2.2 (−5.3, 10.2)
	NO_2_	4.4 (−2.7, 12.0)	4.4 (−3.5, 13.1)	2.4 (−4.3, 9.7)
	CO	1.2 (−11.8, 16.0)	0.6 (−14.8, 18.7)	−0.3 (−13.3, 14.5)
	O_3_	−0.5 (−4.2, 3.4)	−1.4 (−5.5, 3.0)	−1.7 (−5.5, 2.2)
Females (35–54)	Benzene	−13.9 (−36.8, 17.2)	−3.9 (−30.0, 31.8)	6.2 (−18.6, 38.7)
	PM_10_	−15.8 (−32.8, 5.6)	−17.9 (−35.7, 4.7)	−13.7 (−30.1, 6.5)
	NO_2_	−15.1 (−29.5, 2.3)	−12.9 (−29.0, 6.8)	−5.9 (−21.4, 12.6)
	CO	−30.5 (−54.2, 5.3)	−14.3 (−46.1, 36.3)	11.9 (−23.9, 64.5)
	O_3_	−5.7 (−15.7, 5.5)	−8.5 (−19.4, 3.9)	−8.5 (−18.6, 2.9)
Females (55–74)	Benzene	21.5 (4.6, 41.0)*	29.6 (10.3, 52.2)**	27.1 (9.8, 47.1)**
	PM_10_	9.5 (−3.1, 23.9)	16.8 (2.0, 33.7)*	17.8 (4.2, 33.1)*
	NO_2_	7.2 (−4.6, 20.5)	15.0 (0.9, 31.2)*	15.4 (3.0, 29.3)*
	CO	9.1 (−11.6, 34.6)	22.1 (−5.5, 57.8)	23.3 (−1.7, 54.6)
	O_3_	−3.8 (−10.1, 2.9)	−6.0 (−13.0, 1.5)	−5.9 (−12.2, 0.9)

a Associations observed for different lag times; excess odds ratios (eOR) are expressed as per cent (95% confidence interval) increase for *i*) a 1 µg/m^3^ increase in benzene concentrations; *ii*) a 10 µg/m^3^ in NO_2_, O_3_ and PM_10_ concentrations and *iii*) a 100 µg/m^3^ increase in CO concentrations. Adjusted for the previous day maximum atmospheric pressure, same day minimum temperature and influenza epidemics.

*p<0.05.

**p<0.001.

As regards to the effects of other ambient air pollutants, we have found a higher risk for younger men for NO_2_ (lag 0, eOR = 9.3 [0–19.4]). In older women (55–74 years), NO_2_ was strongly associated with MI risk for lag 0–1 (eOR = 15.0 [0.9–31.2]) and lag 1 (eOR = 15.4 [3–29.3]), as were PM_10_ (lag 0–1, eOR = 16.8 [2–33.7]; lag 1, eOR = 17.8 [4.2–33.1]). Models considering the May-September period for ozone were very similar to those covering the April-September period. Incorporating mean relative humidity and holidays as covariates led to results very similar to the above. In addition, influenza epidemics did not influence the results.

## Discussion

Our finding of an association between ambient benzene and the triggering of MI has never been reported before. A possible explanation is that benzene is inconsistently measured in standard air monitoring systems, and so far essentially associated to long-term effects, such as cancer.

Among the strengths of our work is the accurate air pollution modeling at a very fine scale (census block) over the study period, diminishing as much as possible the potential for exposure misclassification. Another robust feature is case collection from a specialized registry, using internationally validated diagnosis ascertainment procedures. In addition, using a proven, robust case-crossover design, we observed associations (Table 4) between classically studied traffic-related pollutants (PM_10_, NO_2_, CO and ozone) and outcomes that were within the range reported in the literature [3], although non-significant for the study population as a whole, due to our limited sample size. Nonetheless, it appears a striking effect of benzene. However, this observation could be partially confounded by ultrafine particulate matter [21], unmeasured in this study.

Ambient air pollutants, in particular those produced by traffic, are systematically found being highly correlated. This is the case for PM_10_ and PM_2.5_, the latter (unmeasured in our study) being a sizeable constituent of the PM_10_ fraction [22]. In addition, unmeasured pollutants may confound associations [23]. Available methods aiming at disentangling the separate effects of individual pollutants do not provide so far a gold standard [24] and results remain difficult to interpret [25]. As most other authors, we assessed excess risks for each pollutant under study.

We observed the expected [2], [26] baseline risk differences between genders, with a 3.3/1 male/female ratio in our study population. In subgroups analysis, younger males appear more at risk (benzene and NO_2_, lag 0) than older ones, perhaps because the conditions or cumulative risk factors that contribute to a MI in this age group make them especially sensitive, to effects of benzene in particular. Older females appear also at higher risk with benzene, and with NO_2_ and PM_10_ (Table 5). Such results have already been reported in the literature for the latter two pollutants [23], [27] although not convincingly explained so far.

No data were available at the individual level on tobacco smoking and lifestyle, but these factors contribute concurrently to long term susceptibility, not to the very short-term circumstances triggering a MI.

Altogether, the overall consistency between our results and those published in the literature, associating exposure to usual traffic-related air pollutants and the triggering of MI, lends support to our finding of an association between this outcome and exposure to benzene.

As for limitations of this study, we acknowledge that there remains some room for exposure misclassification, since exposure was assimilated to the levels of air pollutants in the subjects' census block of residence. We have no data on the mobility of our study population. However, the lags showing a positive, statistically significant association between benzene exposure and MI onset span from the same day to the previous day. Thus, exposure misclassification as regards time spent out of area of residence is limited, since people usually spend the major part of their time at home. We feel highly unlikely that such relative misclassification could account for the sizeable associations we have observed.

### Mechanisms of action

The literature extensively addresses the underlying mechanisms of action of ambient air pollutants involved in the triggering of MI. Overall, it appears that changes in the synthesis or reactivity of nitric oxide that may be caused by environmental oxidants [28] or an increased endogenous production of reactive oxygen species are candidate mechanisms [29]. A recent review (although targeted to benzene-induced mutation mechanisms) indicates that oxidative stress is one mechanism-of-action of benzene as well [30], but the real contribution of such mechanism to the association we have observed remains to be assessed. In addition, short-term associations with ambient benzene have also been shown for asthma exacerbation [18], although providing no clues for a mechanism of action. The development of epigenetics may shed some light on intimate mechanisms [31].

### Public health impact

In the above cited meta-analysis [3], the authors estimated the population attributable fractions (PAF) for those pollutants. Assuming a 100% prevalence of exposure, the PAFs were of 4.5% for an incremental exposure of 1 mg/m^3^ carbon monoxide, and ranging between 0.6% and 2.5% for a 10 µg/m^3^ incremental exposure to the other pollutants. In an earlier analysis of the relative importance of triggers of MI [32], calculated from 14 studies an overall PAF for air pollution of 7.4%. That is, such PAF is of a magnitude similar to that of other well-documented triggers such as physical exertion, alcohol, and coffee. If the whole community were exposed, such a relatively limited PAF would have considerable public health impact. Provided our findings were replicated, this would be the case for benzene but with a much higher effect. However, we felt that calculating a PAF for benzene would be irrelevant in the absence of convergent studies.

## Conclusion

We have observed a benzene-associated risk for the triggering of myocardial infarction, using a robust characterization of cases and of exposure. This association has not been documented previously. In addition, the strength of the association was greater for benzene as compared to traffic-related pollutants usually investigated, such as particulate matter. Of course, these results may be the product, of an unmeasured confounder (at least partially for ultrafine particles, which are strongly correlated to PM_10_). If our findings were confirmed by others, this would mean that not only diesel vehicles, the main particulate matter emitters [21], [33] but also gasoline-fueled cars –main benzene emitters-, should be taken into account for public health action.
